# Supplementary material for: PHENSIM: Phenotype Simulator
Source: PLoS Comput Biol. 2021 Jun 24;17(6):e1009069. doi: 10.1371/journal.pcbi.1009069 (PMC8224893; doi:10.1371/journal.pcbi.1009069)
Supplement: S1 Text — (DOCX) [file pcbi.1009069.s004.docx]

# S1 Text. Supplementary Results

## Simulation #1: Anti-cancer effects of metformin

Metformin is an agent for the treatment of type 2 diabetes [1-4]. It inhibits glucose production in the liver and increases insulin sensitivity in the peripheral tissues, resulting in elevated glucose uptake and skeletal muscle and adipose tissue consumption. Metformin treatment reduces insulin secretion by β-pancreatic cells. The key molecule that performs these functions is AMP-activated protein kinase (AMPK), a serine-threonine kinase regulating cellular energy metabolism.

Several evidence has indicated that metformin possesses anti-cancer effects in various cancer types, especially in diabetic patients, directly and indirectly [1-3]. Indeed, metformin directly activates the LBK1-AMPK signaling pathway [3]. Metformin is known to uncouple the electron transport chain in the mitochondria by targeting Complex I ([1, 2, 5]), leading to impaired mitochondrial function, decreased adenosine triphosphate (ATP) synthesis, and elevated cellular AMP/ATP ratio [1, 3]. Increased AMP binding to AMPK activates AMPK by inducing phosphorylation of its catalytic subunit at residue Thr172 by liver kinase B1 (LKB1), a tumor suppressor and a regulator of cellular energy status [2, 3]. The binding of AMP to AMPK also prevents the dephosphorylation of AMPK Thr172 by protein phosphatases. LKB1-activated AMPK phosphorylates and activates the tumor suppressor Tuberous Sclerosis Complex 1 and 2 (TSC1/2), which negatively regulates the activity of the mammalian target of rapamycin (mTOR), which is upregulated in most cancer cells and causes tumor proliferation and cell growth by inhibiting Ras homolog enriched in brain (Rheb) [1, 3]. mTOR is a critical mediator of the phosphatidylinositol-3-kinase/protein kinase B/Akt (PI3K/PKB/Akt) signaling pathway, one of the most frequently deregulated molecular networks in human cancer [3].

Metformin-activated AMPK inhibits mTOR and reduces the phosphorylation of its downstream targets, the eukaryotic initiation factor 4E-binding proteins (4EBPs), and ribosomal S6 kinases (S6Ks), leading to an inhibition of global protein synthesis, cell cycle progression, cell proliferation, and angiogenesis [3]. Moreover, AMPK has been reported to suppress the mTOR signaling pathway independent of TSC2 via phosphorylation of mTOR binding protein Raptor.

Metformin has been shown to cause a G0/G1 cell cycle arrest by decreasing the expression of cyclin D1 [2].

Metformin-induced AMPK activation has been shown to phosphorylate insulin receptor substrate-1 (IRS-1) at Ser-794, which results in decreased recruitment of the p85 subunit of phosphoinositide-3-kinase (PI3K), thus, impairing the insulin-like growth factor (IGF)-stimulated PI3K/protein kinase B/ mammalian target of rapamycin complex 1 (PI3K/Akt/mTORC1) signaling pathway.

Metformin also inhibits the crosstalk between G-protein-coupled receptors (GPCR) and insulin/IGF1 receptors signaling, resulting in the inhibition of mTORC1 and reduction of cellular proliferation [1, 2].

Metformin induces nuclear degradation and decreased expression of Sp proteins, transcription factors for genes involved in cell proliferation (cyclin D1), metabolism (FAS), apoptosis (B-cell lymphoma 2, BCL-2, and survivin), and angiogenesis (vascular endothelial growth factor, VEGF, and its receptor VEGFR1) [2, 3].

The indirect mechanism of metformin in anti-cancer function is related to its ability to lower insulin and insulin-like growth factor 1 (IGF-1) [3].

Metformin disrupts insulin and IGF-1 signaling pathways by reducing insulin and IGF-1 levels, reducing total IGF-1 receptor and IR levels, and downregulating IGF-1 receptor and IR gene expression [6].

In parallel with this, metformin also downregulates the MAPK (mitogen-activated protein kinase) pathway, NF-𝜅B (nuclear factor kappa B) signaling [5, 7], glycolysis, and the TCA (tricarboxylic acid) cycle [2, 3, 6].

Based on these details, we run a PHENSIM simulation of the simultaneous upregulation of LKB1 and the downregulation of insulin (Ins), IGF1, and GPD1 [8].

As expected, PHENSIM returned significant downregulation of Insulin (pathway activity score = -8.7121, p-value 0.105) and mTOR signaling (pathway activity score = -8.7121, p-value 0.107).

Although mTOR's negative regulation should activate the *repressor of translation initiation* 4EBP, the simulation returns no activity score for this node. However, a low positive perturbation for 4EBP can be observed (perturbation = 0.00009). PHENSIM also predicted the inhibition of downstream nodes involved in protein synthesis, such as S6Ks (S3A Fig) (S6K-alpha3 activity score = -2.0019, p-value = 0.046).

MAPK signaling was predicted as downregulated (MAPK pathway activity score = -4.8203, p-value = 0.130). Several downregulated enzymes and metabolites were predicted for these two pathways, in full agreement with data from literature [6] (S3B Fig and S3 Table).

Finally, in accordance with literature, PHENSIM also predicted weak changes in cytokine gene expression as it can be seen from average nodes perturbations (IL6 perturbation = -0.0001; IL8 perturbation = -0.00002; IL17 perturbation = -0.00001; TNF-alpha perturbation = -0.00014) [6].

## Simulation #2: Everolimus (RAD001) and breast cancer

Everolimus (RAD001, Afinitor®), an analog of rapamycin, has shown immunosuppressive and anti-cancer activities [9-12]. It is currently approved to treat various cancer types, including metastatic breast cancer [13-15]. Everolimus has a growth inhibitory activity against tumor cells and can retard tumor growth through direct mechanisms against both the tumor cell and the solid tumor stromal components [11].

Everolimus inhibits the “*mammalian target of rapamycin”* (mTOR) to prevent the downstream signaling required for cell cycle progression, cell growth, and proliferation [9-11, 16-18].

In mammalian cells, mTOR exists in two complexes, mTORC1 and mTORC2 [14, 15, 17, 19], which are differentially regulated and have distinct substrate specificities [14]. mTORC2 signaling is lower in breast tumors compared to normal breast tissue. This difference could suggest that mTORC1 signaling is more oncogenic than mTORC2 [20].

mTORC1 promotes protein synthesis by (a) stimulating ribosome biogenesis via phosphorylation and inhibition of the RNA Polymerase III repressor MAF1 [21]; (b) phosphorylating the p70S6K and 4EBP1 and modulating the activity of their downstream targets [22, 23]; (c) by regulating nucleocytoplasmic RNA transport [21, 22]. In addition, mTORC1 stimulates pyrimidine biosynthesis and lipid biosynthesis [22, 23]. mTORC1 phosphorylates ULK1 (unc-51 like autophagy activating kinase 1) and DAP (death-associated protein) inhibiting autophagy [20, 24].

Finally, the upregulation of mTOR signaling can promote tumor growth and progression through several mechanisms, including the promotion of growth factor receptor signaling, angiogenesis, glycolytic metabolism, lipid metabolism, cancer cell migration, and suppression of autophagy [14, 15]. All these functions of mTORC1 are reversed by Everolimus and other mTORC1 inhibitors [10, 13] (S7 Fig).

Everolimus binds with high affinity to its intra­cellular receptor, the FKBP12, a protein belonging to the immu­nophilin family. The Everolimus–FKBP12 complex binds mTOR when associated with RAPTOR and mLST8 to form mTORC1 complex, resulting in decreased interaction between mTOR and RAPTOR, which could inhibit the phosphorylation and activation of the major mTORC1 downstream targets [12, 14-16, 19, 20].

Here we wanted to simulate the inhibition of mTORC1. Unfortunately, simulating mTORC1 inhibition was not feasible because KEGG does not distinguish the mTOR node in mTORC1 from the one included in mTORC2. To overcome such limitation, we have set the downregulation of p70S6K (p70S6Ka and p70S6Kb) and 4EBP and the upregulation of ULK1/2 because these are the well-known downstream targets of mTORC1. Then we uploaded a list of non-expressed genes in breast tissue to simulate the drug’s effects on such tissue. Our simulation predicted that RNA transport factors would be downregulated, while factors involved in autophagy would be upregulated. The simulation showed that RNA transport signaling pathway exhibits a negative activity scores (activity score = -4.4108; p-value = 0.13) (S8A Fig). Furthermore, we could predict several downregulated factors involved in RNA transport and protein synthesis, such as eukaryotic translation initiation factor 4A, 4B and ribosomal proteins S6Ks, p70-S6K and p70S6Kb (eIF4A1 activity score = -4.8203; eIF4A2 activity score = -4.8203; p70-S6K activity score = -4.8203; p70S6Kb activity score = -4.8203; p-value for all nodes < 0.01). PHENSIM also predicts the 4EBP1 inhibition (activity score = -4.8203; p-value = 0.013) and consequently the upregulation of eIF4E (activity score = 4.8203; p-value = 0.008) (S8B Fig).

PHENSIM predicts upregulation of the autophagy (activity score = 4.8203, p-value = 0.26) as a consequence of alterations in ULK1/2 phosphorylation levels and the downregulation of cyclin D. However, PHENSIM failed in predicting the deregulation of p21 (cyclin-dependent kinase inhibitor 1) and NF-kB [13]. This limitation is probably due to the presence of a single node for mTORC1 and mTORC2.

## Simulation #3: effects of exosomal vesicles on hematopoietic stem/progenitor cells (HSPCs) in the bone marrow (BM)

Cancer-derived exosomes’ functional relevance to tumor growth, metastasis, and treatment response has become increasingly evident [25, 26]. Exosomes derived from AML blasts contain complex cargoes which function via paracrine mechanisms to modulate the properties of both the tumor cells themselves and the BM niche. Several microRNAs are selectively incorporated in these exosomes, including miR-150 and miR-155 [27, 28]. One of these microRNAs targets is the transcription factor c-MYB, which is downregulated in tumor cells exposed to the exosomes [28]. Additional targets include c-KIT, DNMT1, Lymphoid Cell Helicase (HELLS), PAICS, an enzyme involved in purine biosynthesis, TAB2, and others. The downregulation of these molecules compromises hematopoiesis via stroma-independent mechanisms. However, the cargo of AML cell-derived exosomes also targets mesenchymal stromal progenitors, inhibiting/reducing the expression of hematopoietic stem cell supporting factors such as CXCL12 (C-X-C motif ligand 12), KITL (c-Kit ligand), IL-17, and IGF1 and interfering with both hematopoiesis and osteogenesis [25] (S9 Fig). Moreover, AML-derived exosomes increase gene expression supporting AML growth (DKK1, IL-6, CCL3).

To determine whether PHENSIM can make the correct predictions in this model, we simulated the uptake of the eight most representative miRNAs (miR-150, -155, -146a, -191, -221, -99b, -1246, and let-7a) included in AML-derived exosomes by hematopoietic stem cells [27].

The simulation predicts an inhibition of osteoclast differentiation (activity score = -8.7121, p-value = 0.132) and cytokine-cytokine receptor interaction pathways (activity score = -4.8203, p-value = 0.115) (S9A Fig and S9B Fig).

In agreement with the literature, some genes involved in modulation of normal hematopoiesis, like CXCL12 (activity score = -4.4108, p-value = 0.008) and the receptor IGF1R (activity score = -4.8203, p-value = 0.038), but not IGF1, were downregulated [25]. Similarly, c-MYB, which is involved in HSPC differentiation and proliferation, was also downregulated [28] (activity score = -4.5951; p-value = 0.012) (S10C Fig). However, PHENSIM failed to predict the upregulation of DKK, IL6 and CCL3 (DKK and CCL3 activity score = 0, IL6 activity score = -4.7015, p-value = 0.011), and the downregulation of KITL and IL17 (activity score = 0) [25].

## Simulation #4: testing TNFα/siTPL2-dependent synthetic lethality on a subset of human cancer cell lines

TNFα (tumor necrosis factor alpha), a type II transmembrane protein, is a member of the tumor necrosis factor cytokine superfamily and has an essential role in innate immunity and inflammation.

Although it can induce cell death, most cells are protected by a variety of mechanisms.

In a recent paper, Serebrennikova et al. [29] showed that one of the checkpoints of TNFα-induced cell death is TPL2 (MAP3K8), a MAP3 kinase that is known to have an important role in immunity, inflammation, and oncogenesis. The knockdown of TPL2 resulted in the downregulation of miR-21 and the upregulation of its target CASP8 (caspase-8). This effect, combined with the downregulation of the caspase-8 inhibitor cFLIP (FADD-like IL-1β-converting enzyme inhibitory protein), resulted in the activation of caspase-8 by TNFα and the initiation of apoptosis (fig. 4). The activation of caspase-8 promotes the activation of the mitochondrial pathway of apoptosis. However, some molecules such as BIML (Bcl-2-like protein 11, isoform L), which are also involved in the activation of the mitochondrial pathway, may be activated via caspase-8-independent mechanisms. A crucial upstream regulator of this pathway is NF-κB. The knockdown of TPL2 also inhibits the activation of ERK (MAPK1/2), JNK (c-Jun Nterminal kinase), and p38MAPK, the activation of AKT, and the phosphorylation of GSK3 (glycogen synthase kinase 3) at Ser9/21. However, their inhibition does not appear to have a role in the initiation of TNFα/siTPL2-induced apoptosis. It is worth noting that the activation of the apoptotic (caspase-8-dependent) pathway in TNFα/siTPL2 treated cells was observed in some but not all cancer cell lines, suggesting that correct prediction will depend on whether the data analyzed by PHENSIM are derived from sensitive or resistant cells.

To launch the simulation, we set TPL2 and miR-21-5p as downregulated and TNFα as upregulated. Since our goal was to simulate the outcome of such treatment in six cell lines, i.e., HeLa, HCT116, U2-OS, CaCo-2, RKO, and SW480, we launched six different simulations. Each simulation had a separate list of non-expressed genes, one for each cell line.

Among these tumor cell lines, only HeLa, HCT116, U2-OS were sensitive to treatment with TNFα/siTPL2. At the end of the computations, PHENSIM could not predict the upregulation of caspase-8 for any of the six cell lines neither the downregulation of cFLIP. This limitation could be the result of missing information in KEGG pathways. PHENSIM did not indicate any activity score for MLC1 (Mcl-1 apoptosis regulator) and XIAP (X-linked inhibitor of apoptosis) nodes.

PHENSIM could not predict the upregulation of the apoptosis inhibitors BCL2 and BCL-XL in all cell lines except for HCT116, where BCL2 results positively perturbed (perturbation = 0.001). PHENSIM showed a negative perturbation of the inducer of mitochondrial apoptosis BAX only in HCT116 (S4 Fig).

Although these results do not precisely reflect our expectations as there are discrepancies between the in vitro and in silico experiment done by PHENSIM, it was confirmed by results obtained by the previously mentioned experimental study [29], which suggested that the change in the expression of such molecules was due to the activation of feedback mechanisms.

Besides, phosphorylated ERK, MEK, JNK, and p38 activity were strongly downregulated for all of the six cell lines except for RKO where PHENSIM predict correctly just ERK and p38, and for Caco-2 cells, which result in a negative activity score for ERK and a weak perturbation for JNK and p38 genes. Finally, PHENSIM did not predict cIAP2 (baculoviral IAP repeat containing 2) negative perturbation, which has an activity score of 0 and a weak negative perturbation, in RKO cells as confirmed by the experimental data (S4A Fig and S4B Fig).

# References

1. Bahrambeigi S, Shafiei-Irannejad V. Immune-mediated anti-tumor effects of metformin; targeting metabolic reprogramming of T cells as a new possible mechanism for anti-cancer effects of metformin. Biochemical Pharmacology. 2020;174:113787.

2. Cantoria MJ, Patel H, Boros LG, Meuillet EJ. Metformin and Pancreatic Cancer Metabolism. Pancreatic Cancer-Insights into Molecular Mechanisms and Novel Approaches to Early Detection and Treatment: IntechOpen; 2014.

3. Yu X, Mao W, Zhai Y, Tong C, Liu M, Ma L, et al. Anti-tumor activity of metformin: from metabolic and epigenetic perspectives. Oncotarget. 2017;8(3):5619.

4. Saraei P, Asadi I, Kakar MA, Moradi-Kor N. The beneficial effects of metformin on cancer prevention and therapy: a comprehensive review of recent advances. Cancer management and research. 2019;11:3295.

5. Sekino N, Kano M, Matsumoto Y, Sakata H, Akutsu Y, Hanari N, et al. Antitumor effects of metformin are a result of inhibiting nuclear factor kappa B nuclear translocation in esophageal squamous cell carcinoma. Cancer science. 2018;109(4):1066-74.

6. Gong J, Kelekar G, Shen J, Shen J, Kaur S, Mita M. The expanding role of metformin in cancer: an update on antitumor mechanisms and clinical development. Targeted oncology. 2016;11(4):447-67.

7. Kanigur Sultuybek G, Soydas T, Yenmis G. NF‐κB as the mediator of metformin's effect on ageing and ageing‐related diseases. Clinical and Experimental Pharmacology and Physiology. 2019;46(5):413-22.

8. Schulten H-J. Pleiotropic effects of metformin on cancer. International journal of molecular sciences. 2018;19(10):2850.

9. Citi V, Del Re M, Martelli A, Calderone V, Breschi MC, Danesi R. Phosphorylation of AKT and ERK1/2 and mutations of PIK3CA and PTEN are predictive of breast cancer cell sensitivity to everolimus in vitro. Cancer chemotherapy and pharmacology. 2018;81(4):745-54.

10. Hurvitz SA, Kalous O, Conklin D, Desai AJ, Dering J, Anderson L, et al. In vitro activity of the mTOR inhibitor everolimus, in a large panel of breast cancer cell lines and analysis for predictors of response. Breast cancer research and treatment. 2015;149(3):669-80.

11. O'Reilly T, McSheehy PM. Biomarker development for the clinical activity of the mTOR inhibitor everolimus (RAD001): processes, limitations, and further proposals. Translational oncology. 2010;3(2):65-79.

12. Wazir U, Wazir A, Khanzada ZS, Jiang WG, Sharma AK, Mokbel K. Current state of mTOR targeting in human breast cancer. Cancer Genomics-Proteomics. 2014;11(4):167-74.

13. Lui A, New J, Ogony J, Thomas S, Lewis-Wambi J. Everolimus downregulates estrogen receptor and induces autophagy in aromatase inhibitor-resistant breast cancer cells. BMC cancer. 2016;16(1):1-15.

14. Hua H, Kong Q, Zhang H, Wang J, Luo T, Jiang Y. Targeting mTOR for cancer therapy. Journal of hematology & oncology. 2019;12(1):71.

15. Formisano L, Napolitano F, Rosa R, D’Amato V, Servetto A, Marciano R, et al. Mechanisms of resistance to mTOR inhibitors. Critical Reviews in Oncology/Hematology. 2020;147:102886.

16. Yamanaka K, Petrulionis M, Lin S, Gao C, Galli U, Richter S, et al. Therapeutic potential and adverse events of everolimus for treatment of hepatocellular carcinoma–systematic review and meta‐analysis. Cancer medicine. 2013;2(6):862-71.

17. Royce ME, Osman D. Everolimus in the treatment of metastatic breast cancer. Breast cancer: basic and clinical research. 2015;9:BCBCR. S29268.

18. Kuo C-T, Chen C-L, Li C-C, Huang G-S, Ma W-Y, Hsu W-F, et al. Immunofluorescence can assess the efficacy of mTOR pathway therapeutic agent Everolimus in breast cancer models. Scientific reports. 2019;9(1):1-11.

19. Houghton PJ. Everolimus. Clinical cancer research. 2010;16(5):1368-72.

20. Hare SH, Harvey AJ. mTOR function and therapeutic targeting in breast cancer. American Journal of Cancer Research. 2017;7(3):383.

21. Michels AA. MAF1: a new target of mTORC1. Biochem Soc Trans. 2011;39(2):487-91. Epub 2011/03/25. doi: 10.1042/BST0390487. PubMed PMID: 21428925.

22. Ben-Sahra I, Manning BD. mTORC1 signaling and the metabolic control of cell growth. Current opinion in cell biology. 2017;45:72-82.

23. Ben-Sahra I, Howell JJ, Asara JM, Manning BD. Stimulation of de novo pyrimidine synthesis by growth signaling through mTOR and S6K1. Science. 2013;339(6125):1323-8.

24. Eisenberg-Lerner A, Bialik S, Simon H-U, Kimchi A. Life and death partners: apoptosis, autophagy and the cross-talk between them. Cell Death & Differentiation. 2009;16(7):966-75.

25. Kumar B, Garcia M, Weng L, Jung X, Murakami J, Hu X, et al. Acute myeloid leukemia transforms the bone marrow niche into a leukemia-permissive microenvironment through exosome secretion. Leukemia. 2018;32(3):575-87.

26. Wang X, Chen H, Bai J, He A. MicroRNA: an important regulator in acute myeloid leukemia. Cell Biology International. 2017;41(9):936-45.

27. Hornick NI, Huan J, Doron B, Goloviznina NA, Lapidus J, Chang BH, et al. Serum exosome microRNA as a minimally-invasive early biomarker of AML. Scientific reports. 2015;5:11295.

28. Hornick NI, Doron B, Abdelhamed S, Huan J, Harrington CA, Shen R, et al. AML suppresses hematopoiesis by releasing exosomes that contain microRNAs targeting c-MYB. Science signaling. 2016;9(444):ra88-ra.

29. Serebrennikova OB, Paraskevopoulou MD, Aguado-Fraile E, Taraslia V, Ren W, Thapa G, et al. The combination of TPL2 knockdown and TNFα causes synthetic lethality via caspase-8 activation in human carcinoma cell lines. Proceedings of the National Academy of Sciences. 2019;116(28):14039-48.
