# Supplementary material for: PHENSIM: Phenotype Simulator
Source: PLoS Comput Biol. 2021 Jun 24;17(6):e1009069. doi: 10.1371/journal.pcbi.1009069 (PMC8224893; doi:10.1371/journal.pcbi.1009069)
Supplement: S2 Text — (DOCX) [file pcbi.1009069.s005.docx]

**S2 Text. Stability of the Perturbation Analysis.**

The process leading to the computation of the perturbation is sensitive to the network structure of the meta-pathway. Indeed, as shown in Tarca et al. 2009 [1], equation 3 can be rewritten in matrix form as:

| $PF=\Delta E+W\cdot PF,$ | (S1) |
| --- | --- |

where $\Delta E$ is the column vector of input values computed through equation 2 and W is:

$$W_{n\times n}=\left( \begin{matrix} \frac{w\left( 1,1 \right)}{\sum_{i=1}^{n} w\left( i,1 \right)} & \cdots& \frac{w\left( 1,n \right)}{\sum_{i=1}^{n} w\left( i,n \right)} \\ \vdots& \vdots& \vdots\\ \frac{w\left( n,1 \right)}{\sum_{i=1}^{n} w\left( i,1 \right)} & \cdots& \frac{w\left( n,n \right)}{\sum_{i=1}^{n} w\left( i,n \right)} \end{matrix} \right).$$

The W matrix encodes normalized edge weights. Equation S1 can be rewritten as:

| $\left( I-W \right)\cdot PF=\Delta E.$ | (S2) |
| --- | --- |

Therefore, if $I-W$ is non-singular, the system of equations described by S2 has a solution that can be computed as:

| $\mathrm{PF}=\left( I-W \right)^{-1}\Delta E.$ | (S3) |
| --- | --- |

When the meta-pathway contains feedback loops, the $I-W$ matrix could be singular, leading to an underdetermined system with infinite solutions, leading to a steady increase (decrease) in perturbation for positive (negative) feedbacks without oscillation. However, an estimate of the perturbations could still be obtained using a least-squares approximation of the system S2.

# References

1. Tarca AL, Draghici S, Khatri P, Hassan SS, Mittal P, Kim J-s, et al. A novel signaling pathway impact analysis. Bioinformatics. 2009;25(1):75-82.
