# Supplementary figures and images for: PHENSIM: Phenotype Simulator
Source: PLoS Comput Biol. 2021 Jun 24;17(6):e1009069. doi: 10.1371/journal.pcbi.1009069 (PMC8224893; doi:10.1371/journal.pcbi.1009069)

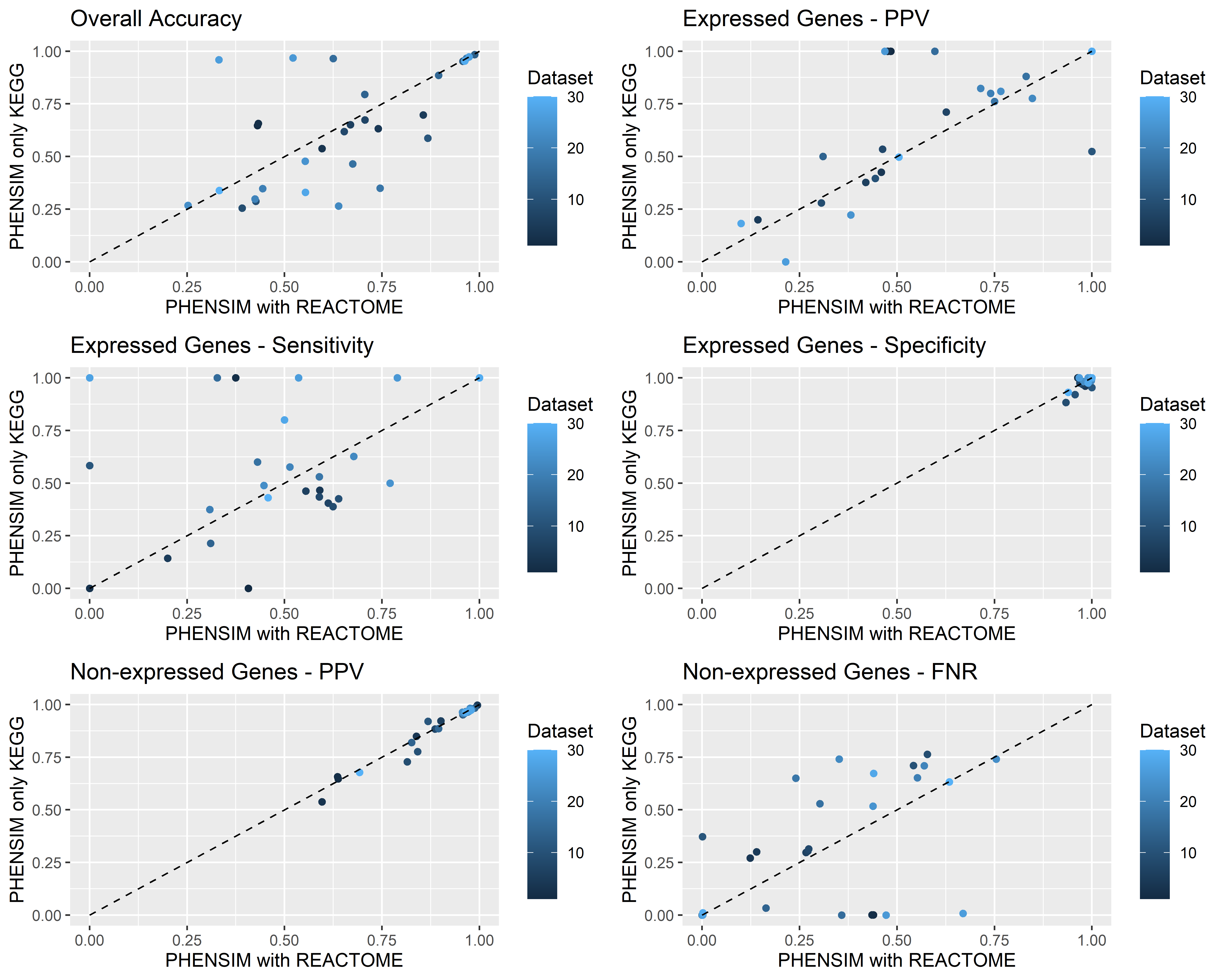

Supplement: S1 Fig — Each graph reports one metric: Positive Predictive Value (PPV), Sensitivity and Specificity for genes showing altered expression, and PPV and False Negative Rate (FNR) for the non-altered ones. On the x-axis, we report PHENSIM performance with REACTOME, while on the y-axis, we present PHENSIM without REACTOME. Each dot is a dataset. The line marks the points where the two variants have the same performance. (TIF) [file pcbi.1009069.s006.tif]

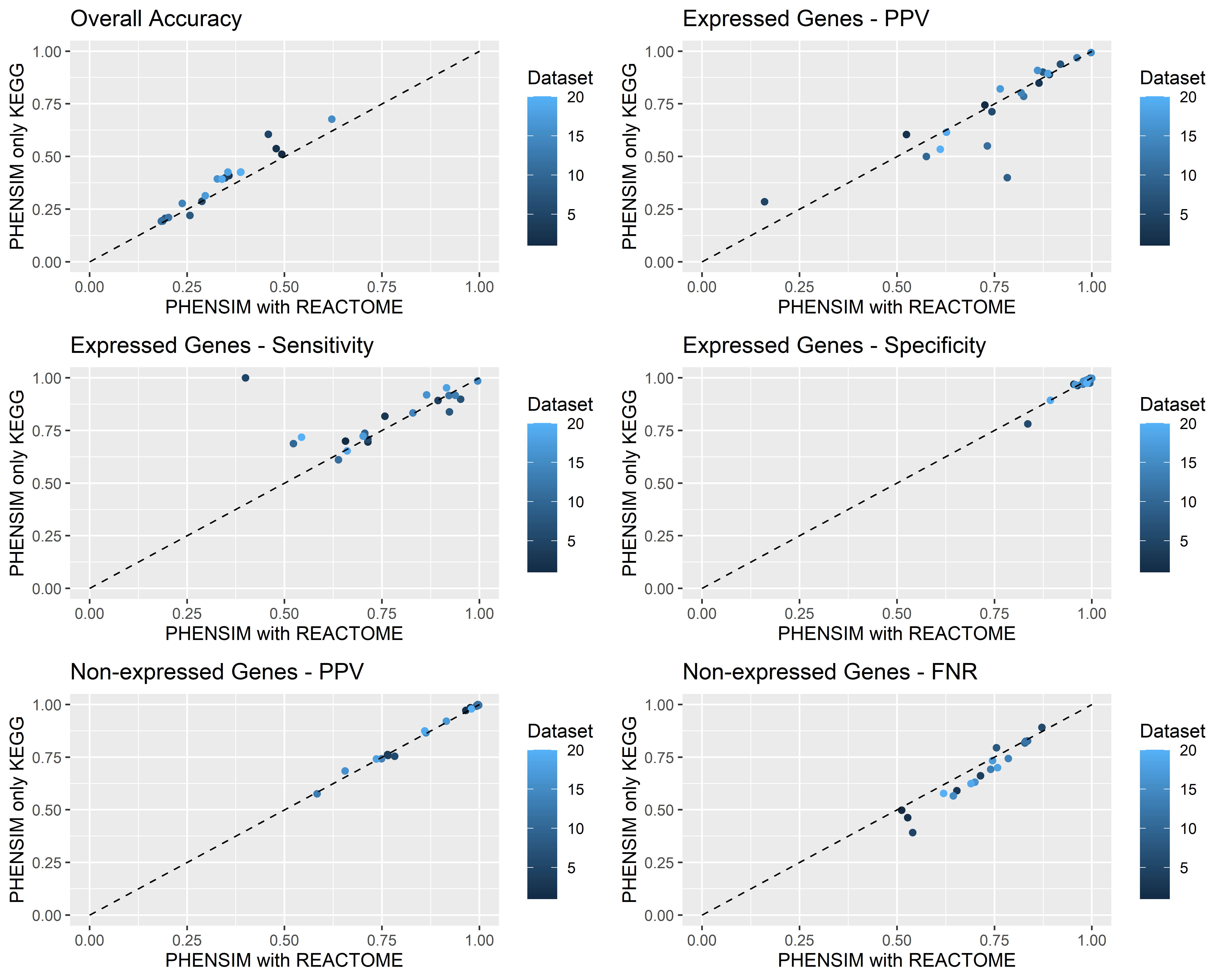

Supplement: S2 Fig — Each graph reports one metric: Positive Predictive Value (PPV), Sensitivity and Specificity for genes showing altered expression, and PPV and False Negative Rate (FNR) for the non-altered ones. On the x-axis, we report the PHENSIM performance with REACTOME, while on the y-axis, we have PHENSIM without REACTOME. Each dot is a dataset. The black line marks the points where the two algorithms have the same performance. (TIF) [file pcbi.1009069.s007.tif]

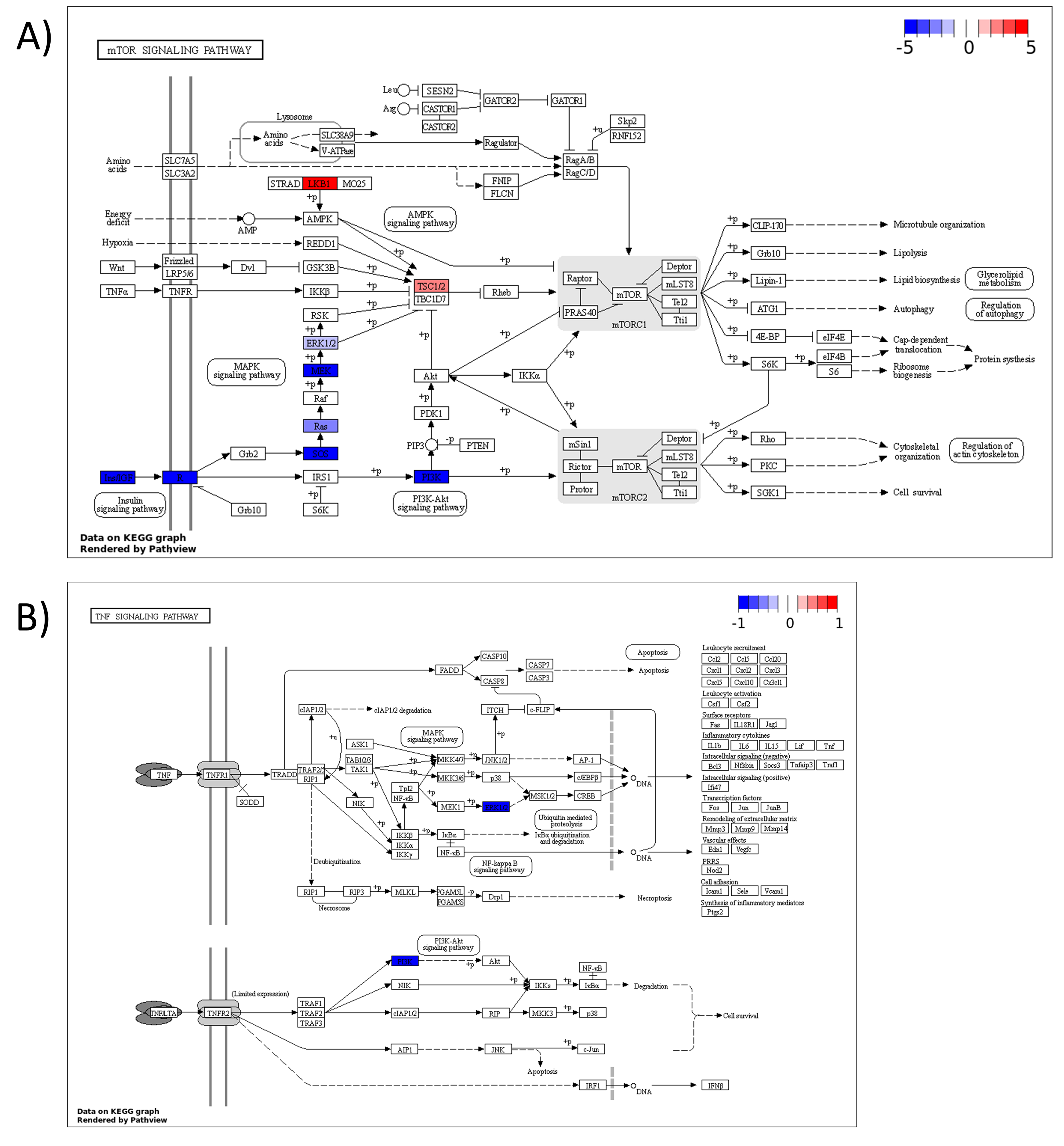

Supplement: S3 Fig — The simulation was launched by assuming the downregulation of INS and IGF-1 and upregulation of LKB1. In S3A Fig, we show predictions related to the mTOR signaling. In S3B Fig, we show predictions related to a subset of nodes belonging to the MAPK signaling and involved in the TNF signaling pathway. Downregulated nodes are colored in blue. Upregulated nodes are colored in red. (TIF) [file pcbi.1009069.s008.tif]

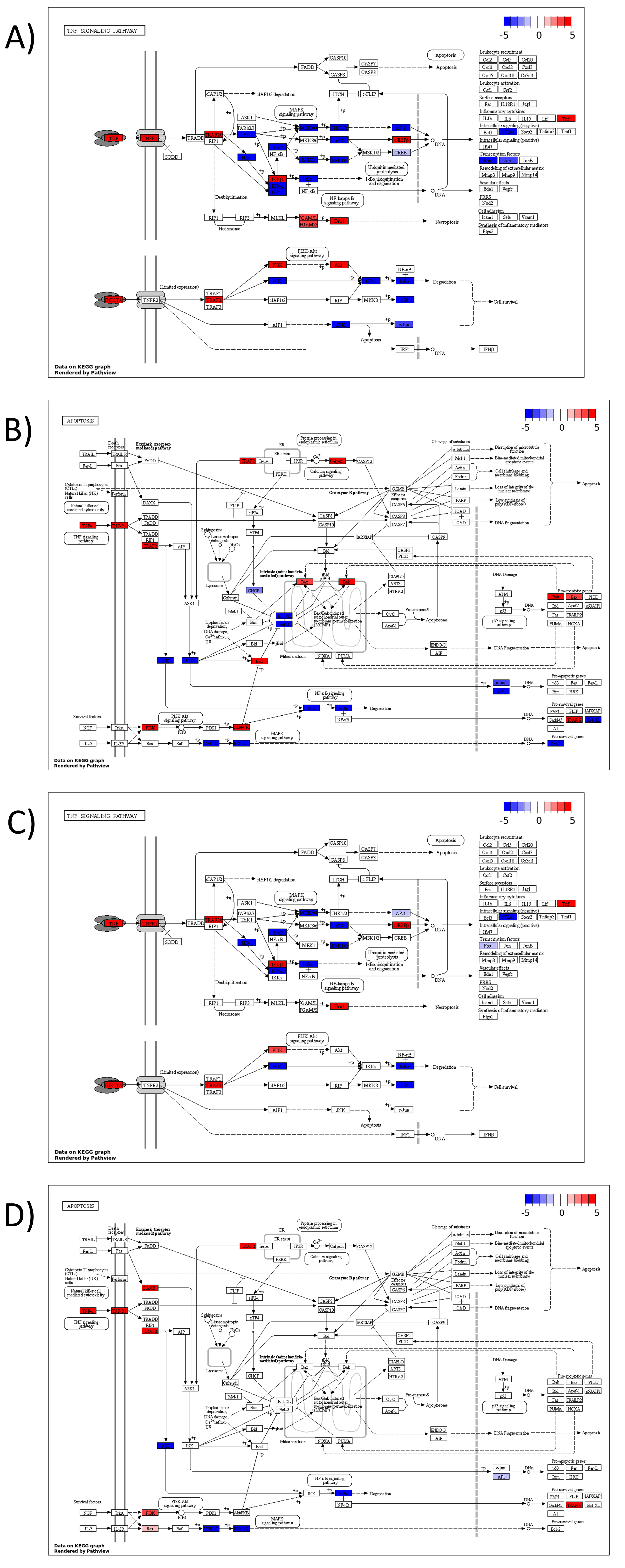

Supplement: S4 Fig — In S4A and S4B Fig are shown results obtained for TNF signaling and Apoptosis pathway, respectively, in the context of HeLa cells (chosen as representative for sensitive cell lines). In S4C and S4D Fig are shown results obtained for TNF signaling and Apoptosis pathway, respectively, in the context of RKO cells (chosen as representative for resistant cell lines). Results for CaCo-2 cells, for which PHENSIM returned a deregulation pattern like that of sensitive cell lines, are not shown. Downregulated nodes are colored in blue. Upregulated nodes are colored in red. (TIF) [file pcbi.1009069.s009.tif]

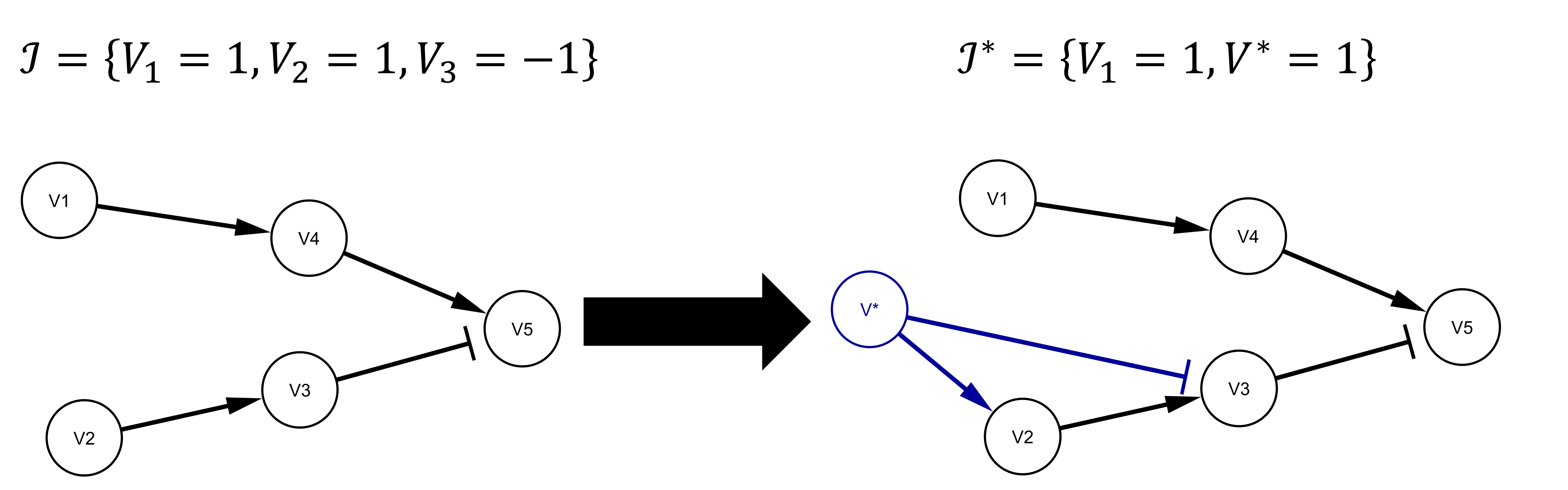

Supplement: S6 Fig — We wish to simulate the upregulation of nodes V1 and V2 and downregulation of V3. Since we know that the expression of V2 and V3 are dependent, we add a novel node V* which activates V2 ((V*, V2) = 1) and inhibits V3 ((X*, X2) = −1). Finally, we can simulate by upregulating both nodes V1 and V*. (TIF) [file pcbi.1009069.s011.tif]

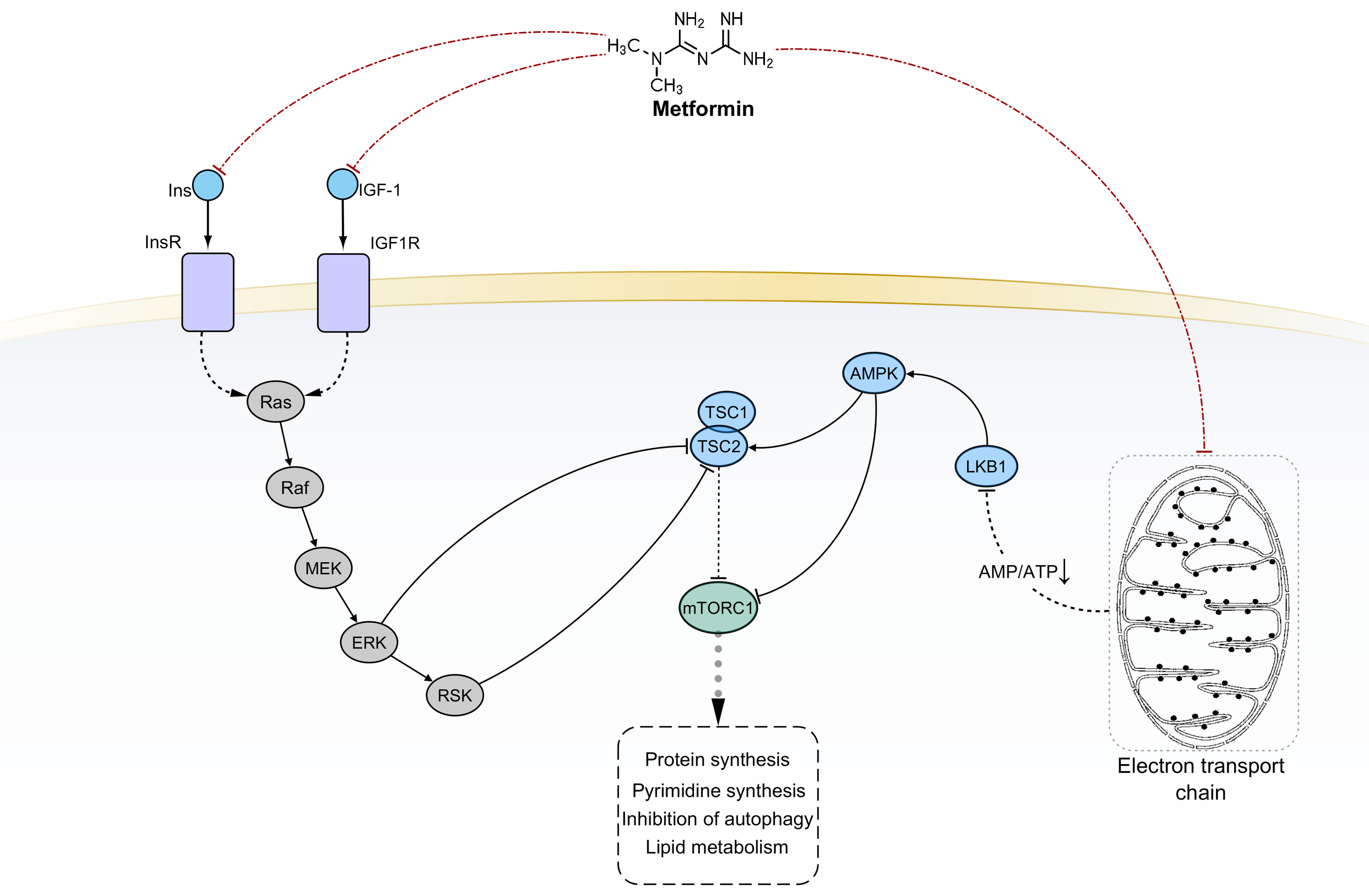

Supplement: S7 Fig — Black solid edges represent direct interaction between first neighbor nodes. Dashed edges represent indirect interactions between nodes. Red dot-dashed edges evidence scientifically validated interactions considered for PHENSIM prediction. (TIF) [file pcbi.1009069.s012.tif]

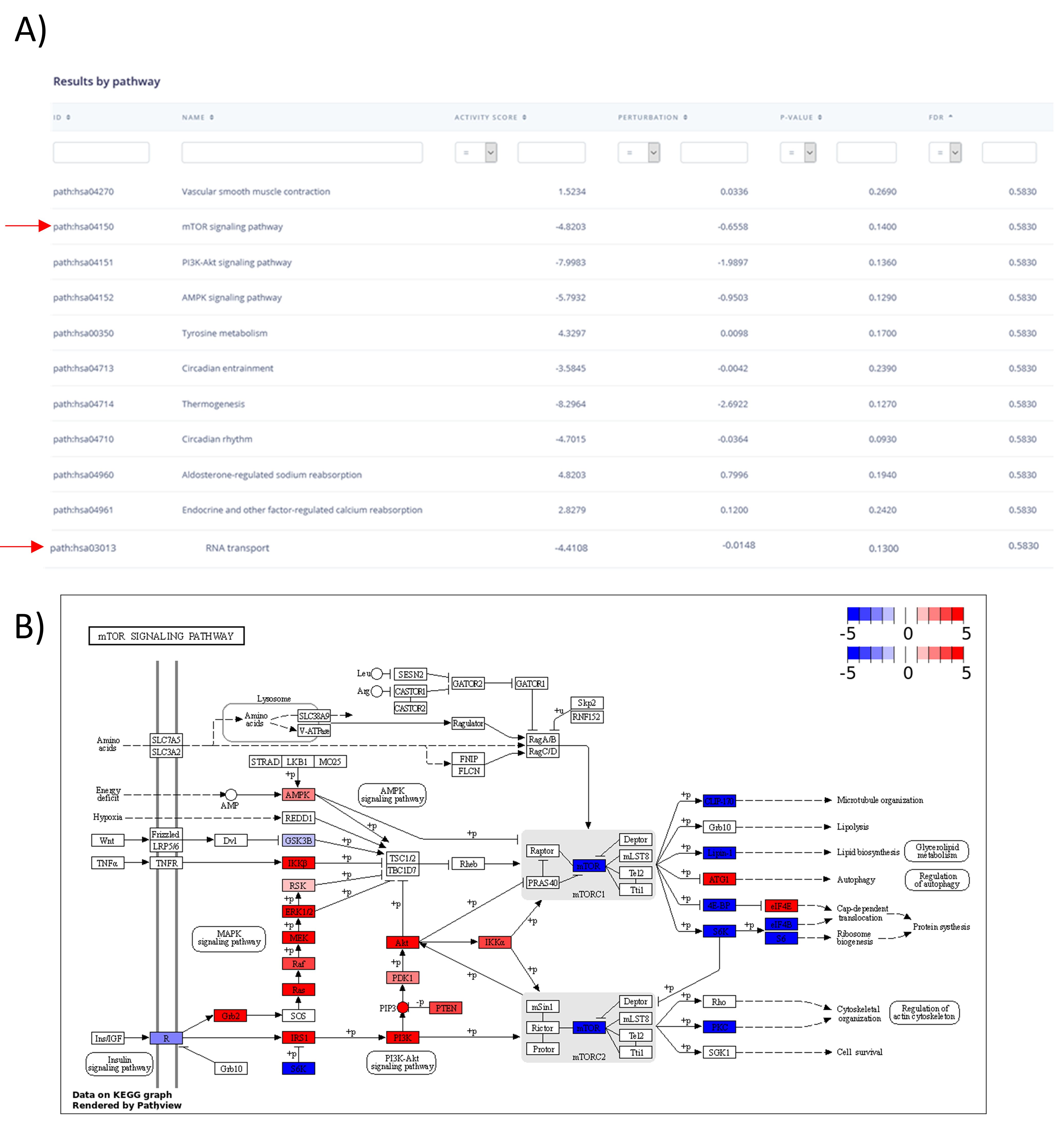

Supplement: S8 Fig — S8A Fig reports the top 10 list of negatively deregulated pathways, among which figured both the RNA transport and mTOR signaling pathways. In S8B Fig are shown predictions related to the mTOR signaling. Downregulated nodes are colored in blue. Upregulated nodes are colored in red. (TIF) [file pcbi.1009069.s013.tif]

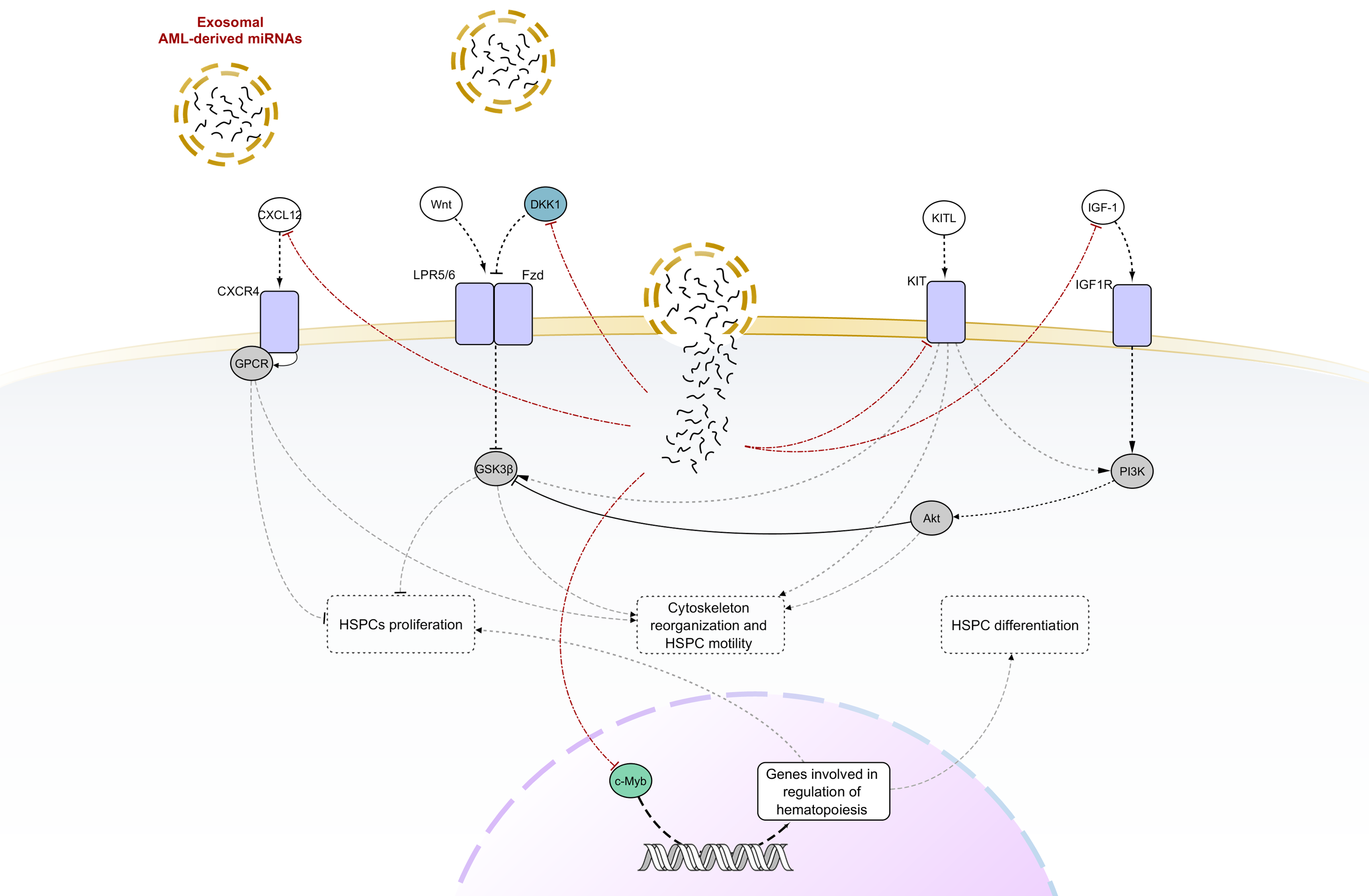

Supplement: S9 Fig — Black solid edges represent direct interaction between first neighbor nodes. Dashed edges represent indirect interactions between nodes. Red dot-dashed edges evidence scientifically validated interactions considered for PHENSIM prediction. (TIF) [file pcbi.1009069.s014.tif]

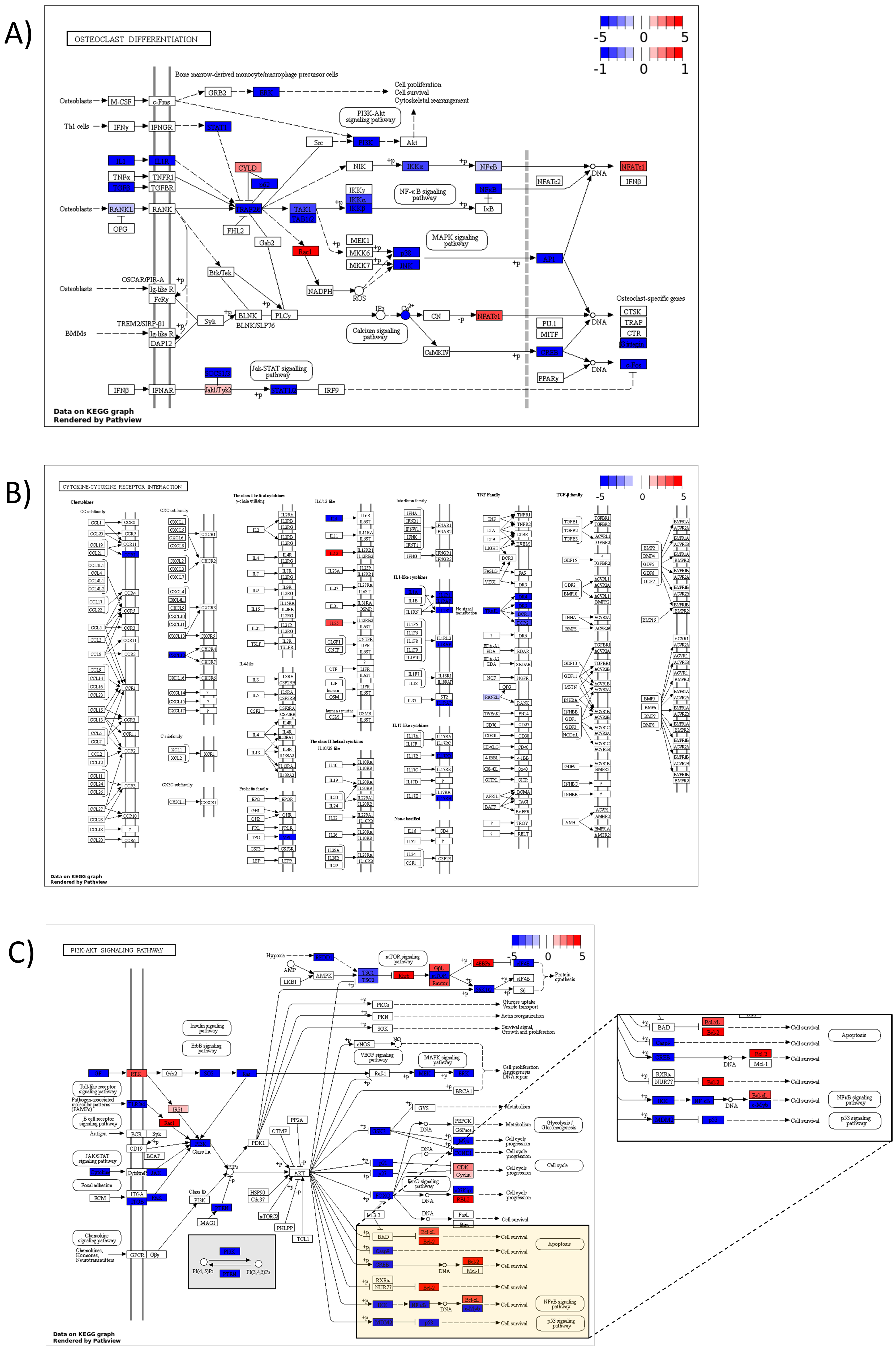

Supplement: S10 Fig — S10A and S10B Fig show the deregulation of several nodes belonging to the Osteoclast differentiation pathway and the Cytokine-cytokine receptor interaction pathway, respectively. In S10C Fig, we show downregulation of c-Myb within the PI3K-Akt signaling pathway. Downregulated nodes are colored in blue. Upregulated nodes are colored in red. (TIF) [file pcbi.1009069.s015.tif]
