# Supplementary material for: PHENSIM: Phenotype Simulator
Source: PLoS Comput Biol. 2021 Jun 24;17(6):e1009069. doi: 10.1371/journal.pcbi.1009069 (PMC8224893; doi:10.1371/journal.pcbi.1009069)
Supplement: Attachment — Submitted filename: Response to reviewers.pdf [file pcbi.1009069.s016.pdf]

Prof. Alfredo Ferro

Reviewer #1

Reviewer question

This manuscript describes a new algorithm for predicting the effects of altered genes, miRNA, and metabolites on KEGGS curated pathways.
This addresses a particularly challenging problem in system biology of predicting the impact of directed (through drug treatment or experiment) or disease dysregulation of pathways. The algorithm is based on the rational of simulating log fold expression changes and its propagation through the network through a weighted sum of up-stream and down-stream connected nodes.
The comparison to BioNSi demonstrates that PHESIM has comparable and even improved performance.

Authors Answer

We thank the reviewer for his essential and valuable comments. It allowed us to redesign our architecture and build a more robust system. Below we provide a point-to-point response to all his remarks.

Reviewer question

My main criticism is that there should be a more quantitative evaluation of the prediction of network perturbations. While the algorithm returns activity score for each node the overall evaluation of the network perturbation relative to expected is left for expert interpretation.
In the four biological examples the authors perform a qualitative assessment of the PHESIM performance based on prior biological knowledge. Admittedly, this is a difficult assessment. One suggestion is to evaluate the algorithm on additional experimental datasets with experimental measurements of gene expression changes following drug treatment or other gene perturbation. For example, PLoS Comput Biol . 2020 Jul 15;16(7):e1007909. doi: 10.1371/journal.pcbi.1007909,
or 10.1371/journal.pcbi.1003290.

Authors Answer

We thank the reviewer for this important suggestion. In our analysis, we wanted to use the four biological examples to indicate what type of experiments could be performed by PHENSIM. We, therefore, chose to evaluate performance using the 22 GEO series datasets as a ground truth that did not require expert interpretation. However, the possibility of

quantitatively evaluating the algorithm on experimental measurements of perturbation experiments is an important test that can highlight our prediction model's consistency with an in vitro model. Therefore, as suggested by the reviewer, we performed the comparison using the most recent dataset (PLoS Comput Biol . 2020 Jul 15;16(7):e1007909. doi: 10.1371/journal.pcbi.1007909) and added the results throughout the manuscript in the Results section and the "Experimental Procedure and Benchmarking" section.

Reviewer question
The authors should also discuss the algorithm's performance in pathways with feedback loops. Specifically, what is the stability of the scores in feedback loops? do they tend to oscillate between activation or suppression? Is the final score sensitive to the number of perturbations performed?

We thank the reviewer for this comment. To better describe how perturbation computation is achieved and the case of feedback loops, we added the "Stability of the Perturbation Analysis" section to the supplementary materials.

Reviewer question
Few additional comments:
- On page 6, a better description of the test data sets is needed. What is "…22 series further divided into 50 case/control sets"? Are these 50 experiments of gene KO experiments? What are the types of perturbations, shRNA, CRISPR?

Authors Answer
The 50 set of samples comes from different types of perturbations. For this reason, we extended Supplementary Table S3 to include details on the kind of experiments. Furthermore, we extended the dataset description in the "Experimental Procedure and Benchmarking" section by adding the following paragraph:

Therefore, we gathered 22 GEO series of cell lines with a perturbed gene. Since these series could contain multiple perturbation experiments of different genes or in several cell lines, we obtained a total of 50 case/control sample sets. Their details are shown in Supplementary Table S3 together with the name and code of the GEO series, the technology used to determine gene expression, the perturbed gene, the type of experiment (knockout, knockdown, transfection, CRISPR, etc.), whether the gene is present in KEGG pathways, and the GEO accessions of the case and control samples.

Reviewer question
- Line 124, page 6, are BioNSi numbers 0.0640, 0.0735? is there extra 0 after decimal?. If true this means BioNSi has no accuracy in these test sets.

Authors Answer

We checked the numbers regarding BioNSi performance. The average accuracy is indeed 0.06401705 for DS1 and 0.07347588 for DS2 (Standard dev.: 0.06582421 and 0.06496027; Minimum: 0.007047478 and 0.009345794; Maximum: 0.2560276 and 0.2334208).

Reviewer question

- It will be helpful to provide additional descriptions of the performance criteria in the context of this work. For example, how is PPV and False Negative Rates computed in this context?

Authors Answer

We extended the section describing the performance criteria by adding the following paragraph:

…we compared PHENSIM predictions (up/down-regulation) with LogFC computed on the expression data. All genes showing an absolute LogFC lower than 0.6 were considered as non-altered. Finally, we assessed the results in terms of accuracy (the number of correctly predicted genes divided by the total number of genes). Furthermore, since accuracy can be influenced by class imbalance, we chose to compute Positive Predictive Value (PPV), Sensitivity, Specificity, and False Negative Rate (FNR) according to the type of alteration found in the expression data. More in detail, for altered genes (LogFC > 0.6), we want to identify upregulation and downregulation events correctly. Therefore, the True Positives (TPs) are genes predicted as upregulated with positive LogFC in the expression data. In contrast, genes predicted as downregulated with a negative LogFC are the True Negatives (TNs). Furthermore, genes predicted as upregulated with a negative LogFC are False Positives, and downregulated genes with a positive LogFC are False Negatives. Now, we can determine the ability of PHENSIM to correctly identify upregulated genes by computing PPV and Sensitivity, while the performance regarding downregulated ones can be assessed through Specificity:

$$PPV = \frac{TP}{TP + FP},$$

$$Sensitivity = \frac{TP}{TP + FN},$$

$$Specificity = \frac{TN}{TN + FP}.$$

Concerning non-altered genes, we are interested in determining whether PHENSIM is capable of correctly identifying them. In this case, a gene that is predicted as non-altered with a LogFC < 0.6 is considered as a True Positive, while a gene indicated as altered with a LogFC < 0.6 is a False Negative. Therefore, we can estimate the rate of correctly identified non-altered genes in terms of PPV, while the FNR shows us the percentage of non-altered genes that are wrongly identified as perturbed by PHENSIM:

$$FNR = \frac{FN}{FN + TP}.$$

Reviewer question
- In the method section, specifically for PHENSIM simulation step and Activity score, it will be helpful to add a graph figure to illustrate the computation (eq. 3 and 4). Similar to Figure S7.

Authors Answer
We thank the reviewer for this comment. We have added a new supplementary figure to explain the computation starting from equation 2 to equation 5. The new supplementary figure replaces Figure S7. Old Figure S7 has been moved to Figure S8.

Reviewer question
- In equation 3, how does the algorithm handle the condition when the sum in the denominator is equal to 0 (i.e the sum of weights of upstream and downstream nodes)?

Authors Answer
We thank the reviewer for the opportunity to clarify this point. In our model, the weight represents the type of interaction between two biological elements in the meta-pathway. Therefore, in our model, the weight is 0 only if no edge is present between the two nodes. Since equation 3 considers all upstream nodes, there must be some connection, and, therefore, the sum of their absolute values will not be 0.

Reviewer question
- The web app is a nice feature of this work. However, the submission UI requires better description. What are the various input fields (nodes) mean? Node is a graph theory term not biological. What is the difference between KO nodes and under-expressed nodes? Are all fields required or only a subset?

Authors Answer
We thank the reviewer for this comment. We have updated the GUI and the user manual to contain more descriptions of all the features. Regarding the difference between KO and under-expressed, we consider under-expressed genes whose expression level is below their "nominal" value (a LogFC lower than zero). On the other hand, KO genes are entirely removed from the pathways and, in principle, cannot be re-expressed by the cell.

Direzione: c/o Presidio Ospedaliero "Garibaldi-Nesima" – U.O. Medicina Interna – via Palermo, 636 – 95122 Catania (CT) – tel. +39.095.759 8401/8402
Direzione e Uffici: c/o Torre Biologica - torre nord, p. 9 - via Santa Sofia n. 89 – 95122 Catania (CT) - tel. +39.095.4781.460/463/464
medclin@unict.it – www.unict.it/medclin

Reviewer #2

Reviewer question
This is an interesting piece of work, which targets a topic with high prospective importance for the bioinformatic community and the system biology research in general. Genome scale models will be more pressingly requested in the coming years, so personally I welcome any work in this field.

Authors Answer
We thank the reviewer for his essential and valuable comments. It allowed us to redesign our architecture and build a more robust system. Below we provide a point-to-point response to all his remarks.

Reviewer question
The tool has been implemented so as to enable easy use and it integrates seamlessly and efficiently the provenance source of KEGG Pathways. However, I think that at its current status, the results that can be generated are of limited importance, primarily because of the multi-tiered architecture of biological networks and the extensive organisational and thermodynamic coupling that governs them.
In that sense I believe that this work would be largely benefit from an effort to integrate more provenance source for cellular networks like Reactome for instance that the authors cite, or even more genome-scale modelling efforts like BioCyc, or network information that could be extracted from massive interaction network data.

Authors Answer
We thank the reviewer for this critical comment. We are aware that KEGG Pathways are limited, but we chose this pathway source since, in our opinion, it is the most reliable one. However, to address this limitation, we added to our model the possibility of using the REACTOME pathway in conjunction with KEGG. We discuss this through the manuscript and assess the model performances with and without REACTOME.

Reviewer question
Another improvement that I would consider largely necessary primarily because it is the advantage of KEGG Pathways that the tool is relying on, is the addition of more supported organisms, as in this way probably the tool would find broader applicability in research communities seeking for similar tools (plant or microbial biologists) than the current ones that the tool targets.

Authors Answer
We thank the reviewer for this important suggestion. We added more species to our user interface. Although the PHENSIM command-line tool supported several species out-of-the-box, the UI required more effort. Now, all supported species can be used directly from

the GUI, and through the GitHub issue system, a user might request the addition of new species if they are available from the pathway databases.

Reviewer question

Also regarding the topic of super pathway modelling there have been seminal efforts in the past that have provided solutions to that (tools like KEGGConverter or KENeV for instance. It is important that the authors cite some of these effort and explain briefly their point of novelty to prior efforts.

Authors Answer

We thank the reviewer for this comment. We discussed this point through the introduction of our manuscript.

Reviewer question

Lastly, it is important that the authors ensured support of interoperability of the networks that their tool is building by supporting export to an XML format like SBML, KGML or RDF format for instance. This could provide larger visibility for the tool and extend its widespread use.

Authors Answer

We thank the reviewer for this suggestion. We revised PHENSIM GUI and added several new features for allowing interoperability with other tools. We allow the download of the simulation results in SBML format for interoperability with System-Biology tools supporting this format and in extended SIF format for Cytoscape and Gephi.
